# Cellobiohydrolase and endoglucanase respond differently to surfactants during the hydrolysis of cellulose

**DOI:** 10.1186/s13068-015-0242-y

**Published:** 2015-03-28

**Authors:** Chia-wen C Hsieh, David Cannella, Henning Jørgensen, Claus Felby, Lisbeth G Thygesen

**Affiliations:** Department of Geosciences and Natural Resource Management, Faculty of Science, University of Copenhagen, Rolighedsvej 23, DK-1958 Frederiksberg C, Denmark; Present address: Center for Bioprocess Engineering, Department of Chemical and Biochemical Engineering, Technical University of Denmark, Søltofts Plads, Building 229, DK-2800 Kgs. Lyngby, Denmark

**Keywords:** PEG, Surfactants, Enzymatic saccharification of cellulose, Monocomponent cellulase hydrolysis, Avicel hydrolysis, PASC hydrolysis, Water constraint

## Abstract

**Background:**

Non-ionic surfactants such as polyethylene glycol (PEG) can increase the glucose yield obtained from enzymatic saccharification of lignocellulosic substrates. Various explanations behind this effect include the ability of PEG to increase the stability of the cellulases, decrease non-productive cellulase adsorption to the substrate, and increase the desorption of enzymes from the substrate. Here, using lignin-free model substrates, we propose that PEG also alters the solvent properties, for example, water, leading the cellulases to increase hydrolysis yields.

**Results:**

The effect of PEG differs for the individual cellulases. During hydrolysis of Avicel and PASC with a processive monocomponent exo-cellulase cellobiohydrolase (CBH) I, the presence of PEG leads to an increase in the final glucose concentration, while PEG caused no change in glucose production with a non-processive endoglucanase (EG). Also, no effect of PEG was seen on the activity of β-glucosidases. While PEG has a small effect on the thermostability of both cellulases, only the activity of CBH I increases with PEG. Using commercial enzyme mixtures, the hydrolysis yields increased with the addition of PEG. In parallel, we observed that the relaxation time of the hydrolysis liquid phase, as measured by LF-NMR, directly correlated with the final glucose yield. PEG was able to boost the glucose production even in highly concentrated solutions of up to 150 g/L of glucose.

**Conclusions:**

The hydrolysis boosting effect of PEG appears to be specific for CBH I. The mechanism could be due to an increase in the apparent activity of the enzyme on the substrate surface. The addition of PEG increases the relaxation time of the liquid-phase water, which from the data presented points towards a mechanism related to PEG-water interactions rather than PEG-protein or PEG-substrate interactions.

## Background

Surfactants have long been added to the hydrolysis step within bioconversion to improve process yields [1-3]. The two most widely used surfactants are polyethylene glycol (PEG) [4,5] and Tween [2,6,7]. When present in concentrations of 0.5 to 5 wt% biomass, these surfactants can increase hydrolysis yields for a given enzyme loading. For example, the addition of PEG improves wheat straw hydrolysis conversion rates by up to 45% [8]. For steam-pretreated spruce, another lignocellulosic substrate, the hydrolysis yield increased 20% with PEG [9]. Experimental evidence has confirmed that PEG increases lignocellulose hydrolysis yields not only via adsorption to lignin to prevent unproductive binding of cellulases, but also by interacting with cellulases to increase their thermal stability [4,5,10]. Other observed mechanisms have been decreased precipitation [11] and subsequent inactivation of the enzymes [12] and by changing the adsorption parameters of cellulases to facilitate enzyme desorption and reduce enzyme loss through irreversible binding [13-15]. Of these mechanisms, the interaction with lignin seems to be the most important from a practical point of view.

Comparing Avicel, corn stover, and bagasse, PEG improved hydrolysis yield mostly for lignin-containing substrates [16]. While there is no doubt that PEG improves hydrolysis of lignin-containing substrates, it has also been shown to boost the hydrolysis of a pure cellulosic substrate, Avicel [9,14,16,17]. Therefore, simple homogeneous model systems consisting of Avicel and PEG can be used as a tool to study the sensitivity of different enzymes to non-substrate-related factors affecting the rate of hydrolysis. In this study, we use such model systems to look into whether individual cellulases are differently affected by addition of PEG and whether the properties of the hydrolysis liquid phase play a role in this effect.

The aim of this work is to present new results on cellulose hydrolysis in the presence of PEG, which we tested using two different monocomponent enzymes, cellobiohydrolase (CBH) I and endoglucanase (EG), as well as two different commercial enzyme preparations. We investigate whether PEG introduces hydrophobic interactions that ‘free up’ water so that it is more available for hydrolysis. Low-field nuclear magnetic resonance (LF-NMR) relaxometry can be used to measure the strength of water-water interactions, and in this paper, we apply LF-NMR to monitor water constraint in the presence or absence of PEG. It has previously been shown that the hydrolysis liquid phase affects hydrolytic enzyme activity [18,19], and in this work, we study whether the effect of PEG on hydrolysis of a pure cellulosic substrate can be linked to a change in water constraint during saccharification.

## Results and discussion

We determined the impact of PEG addition to Avicel hydrolysis using both monocomponent enzymes and commercially available cellulase mixtures. Monocomponent enzyme hydrolysis indicates which cellulases are most affected by the presence of PEG. We also measured the spin-spin (T_2_) relaxation times of the hydrolysis liquid phase at the onset of hydrolysis using LF-NMR for a series of experiments designed to change the water constraint via the presence of PEG and monosaccharides.

### Effect of PEG on different cellulases

**Figure 1:**
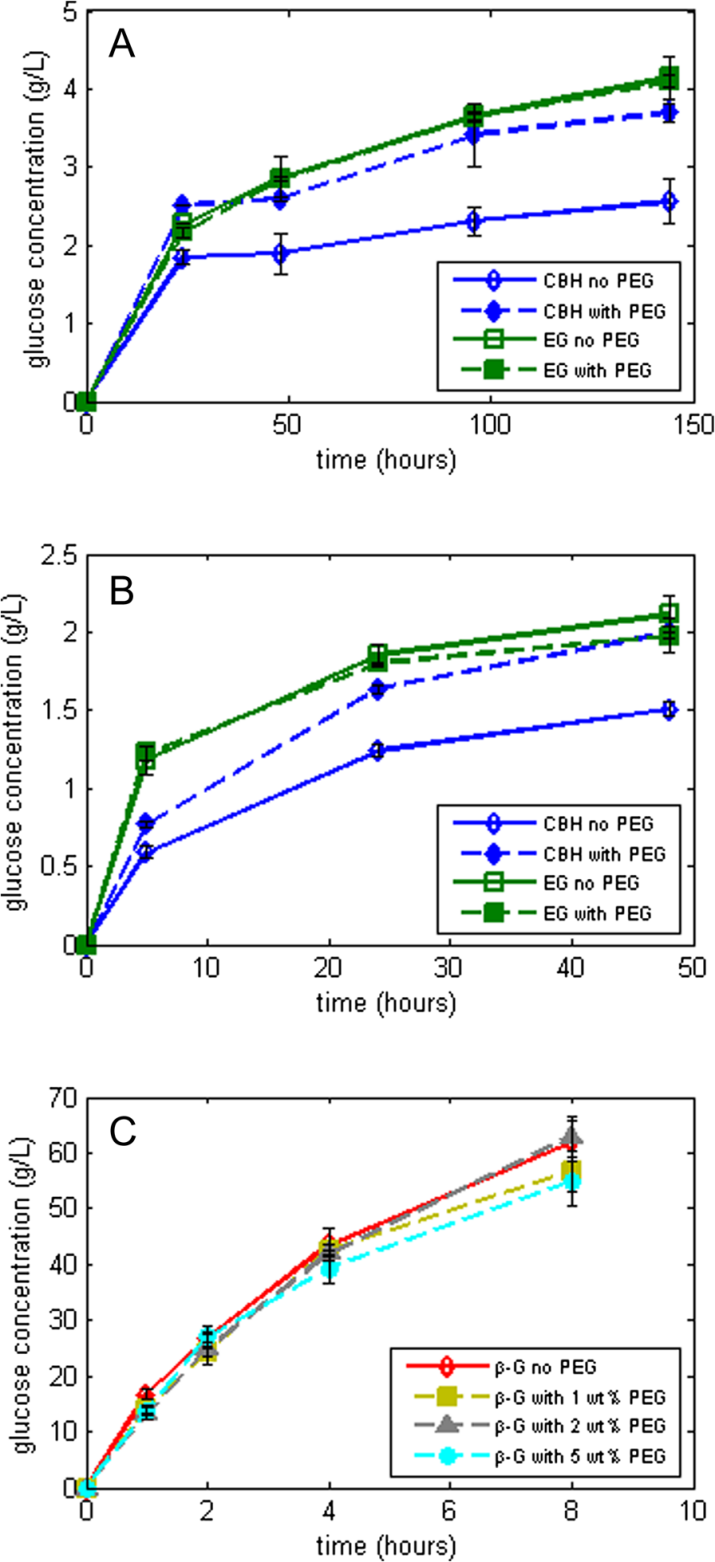
**Effect of PEG on the glucose yields obtained using different substrates. (A)** Hydrolysis of 5% Avicel using monocomponent CBH I and EG with β-glucosidase supplementation in the absence (open symbols) and presence (solid symbols) of PEG 3000, **(B)** hydrolysis of 3% PASC using the same monocomponent enzymes with β-glucosidase supplementation, and **(C)** Hydrolysis of 100-g/L cellobiose using Cellic CTec2 with different concentrations of PEG 3000. CBH, cellobiohydrolase; EG, endoglucanase; PEG, polyethylene glycol.

Previous studies in the literature on the effect of PEG on lignocellulosic biomass used commercially available cellulase preparations [5,9]. The major components of commercial cellulase mixtures are cellobiohydrolases (Cel7A, also known as CBH I) and endoglucanases (EG) [20]. In this study, a monocomponent CBH I and an endoglucanase were tested on Avicel or phosphoric acid swollen cellulose (PASC) in the presence of PEG as shown in Figure 1A,B. The results in Figure 1A show that PEG improved Avicel hydrolysis with CBH I by 45%, while the increase in hydrolysis yield for EG was only 1%. Figure 1B shows that qualitatively similar results were obtained for PASC, confirming that the difference seen is not substrate specific. That is, as a similar difference between hydrolysis with and without PEG is seen for a highly accessible substrate like PASC, the effect of PEG is not to increase substrate accessibility of Avicel for CBH I. While this effect is observed for one specific CBH I and EG obtained from Megazyme (see the ‘Materials and methods’ section), it nevertheless sheds some light on the impact of PEG on cellulases having different hydrolytic mechanisms.

In Figure 1A, at an enzyme loading of 1.2 mg protein/g Avicel, the reaction rate without and with PEG was 77 and 105 mg/L/h, respectively, from time 0 to 24 h, amounting to a 36% difference for CBH I. Towards the end of hydrolysis, that is, from 96 to 144 h, the rate of glucose production without and with PEG is calculated to be 5.2 and 6 mg/L/h, respectively, corresponding to a 15% difference. The main difference, therefore, is observed within the first hours of hydrolysis where PEG plays a larger role in the kinetics of the reaction. We speculate that PEG either increases the activity (*k*_cat_) [21,22] of the enzyme or decreases the *k*_off_ of the CBH I [23-26]. We have also tested different CBH I dosages and observed that the increase in activity was due to the presence of PEG regardless of the dosage of enzyme (data not shown).

The experimental setup shown in Figure 1A,B contained an excess dosage of β-glucosidase in order to avoid end-product inhibition from cellobiose and short-chain oligomers. Thus, the effect of PEG on β-glucosidase activity was also tested by hydrolysis of pure cellobiose with commercially available CTec2. Figure 1C shows little difference between the glucose production with 1, 2, and 5 wt% PEG addition to cellobiose and the control (no PEG). Also, cellobiose was not detected at the final time point in any of the experiments reported in Figure 1, proving that the β-glucosidase dosage was not the limiting factor of the reaction.

That PEG affects CBH I activity only is in agreement with hydrolysis results obtained using another surfactant, Tween 20. Ooshima *et al*. [1] showed that the specific activities of β-glucosidase and endoglucanase were not influenced by 0.05 wt% Tween 20, which was presumed to only enhance the catalytic function of CBH. However, the endoglucanase adsorption to Avicel decreased in the presence of Tween, and its activity in solution increased. Hence, Tween was able to make the endoglucanases desorb more easily from the substrate (increase the *k*_off_) and retain their activity in the solution longer. The mechanism of surfactant addition was then believed to balance the adsorption profiles of exo- and endo-cellulases on the substrate and, hence, increase hydrolysis. Park *et al*. [13] also reported non-ionic surfactants in general to aid in the desorption of cellulases tightly adsorbed to the substrate surface, increasing both the amount of free cellulases in solution and conversion yields. Our experiment was only designed to look at enzymatic activity based on hydrolysis yields, and for this parameter, no significant effect of PEG addition was found for the endoglucanase tested. From these experiments, we can only speculate on the influence of PEG on *k*_off_ as the adsorption profiles of the enzymes were not monitored over time.

### Effect of PEG on enzyme thermal stability

**Figure 2:**
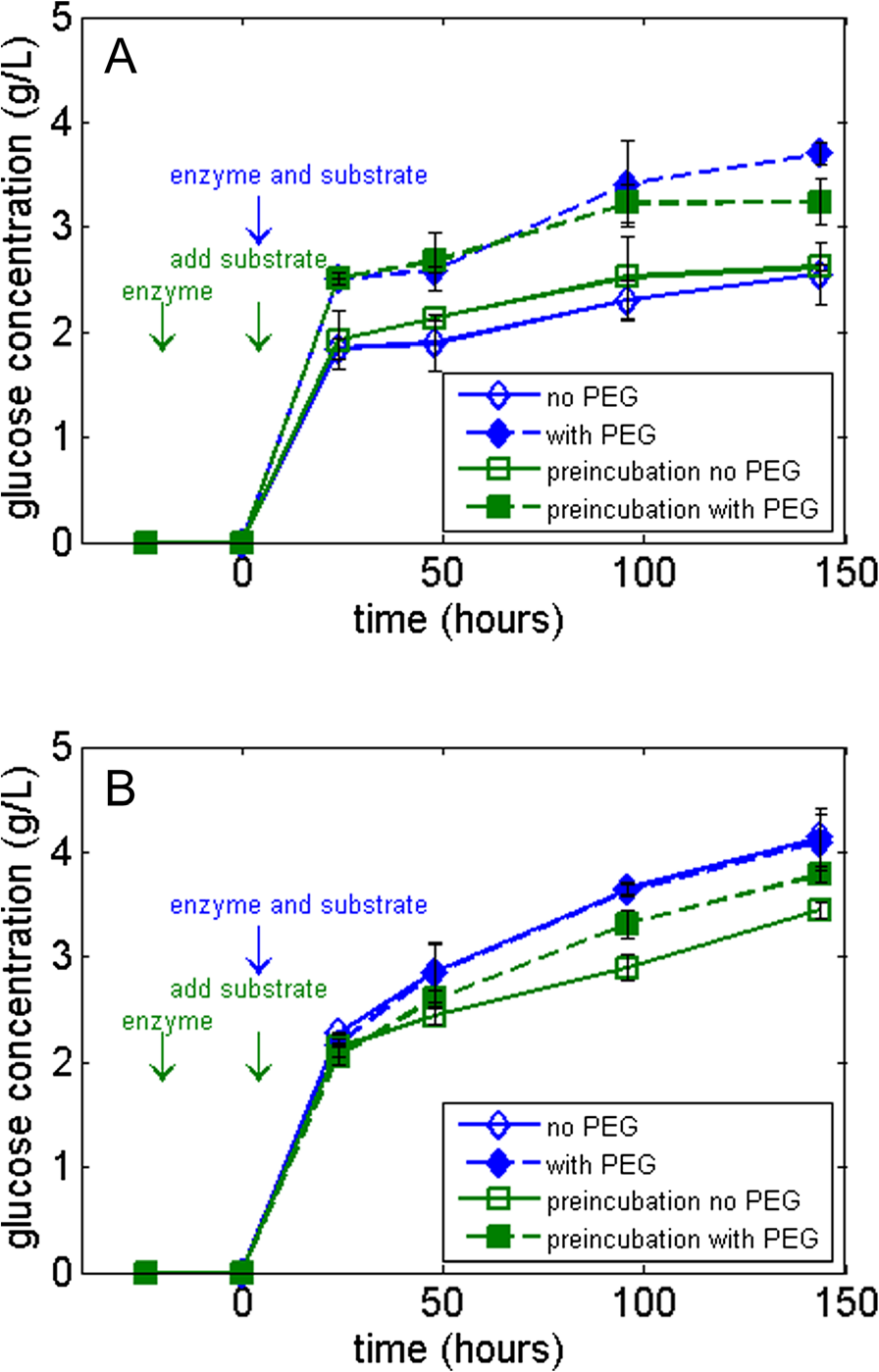
**Thermal stability of monocomponent cellulases.** Hydrolysis of 5% Avicel at 50°C using **(A)** CBH I and **(B)** EG with β-glucosidase supplementation in the absence (open symbols) and presence (solid symbols) of PEG 3000. PEG, polyethylene glycol.

Another way in which PEG may enhance the overall activity of cellulases is by increasing the stability of the enzyme. Reese [12] showed increased stability of cellulases against changes in pH, temperature, shaking, and other environmental factors with the addition of PEG, and Chylenski *et al*. [11] have shown that PEG can prevent enzyme precipitation in solution. We tested the thermostability of both the CBH I and the EG by measuring its retention of activity after incubating the enzymes (without substrate) in buffer at 50°C for 24 h [27], and the results are shown in Figure 2A for the CBH I and Figure 2B for the EG. Figure 2A shows that there was no significant change in the glucose production after CBH I pre-incubation at 50°C. With the presence of PEG, the enzyme activity increased as expected, but the effect of heat treatment on the enzyme activity can be seen only in the later stages of hydrolysis (after 96 h) where yields drop by 14% if the CBH I was subject to a pre-incubation period. For the initial stages of hydrolysis, however, the pre-incubated CBH I yields similar, if not better, glucose yields than those without heat treatment. Thus, it is inconclusive whether PEG has a true effect on the stability of CBH I. As for the EG activity shown in Figure 2B, PEG is shown to stabilize the enzyme after 24 h of hydrolysis after undergoing a 24-h pre-incubation period, increasing the yield 10%. Thus, PEG was able to stabilize the EG against denaturation, verifying that the substantial increase in hydrolysis yield seen for CBH I but not for EG when PEG was added (Figure 1A,B) does not have to do with better thermal stability. CBH I acting on Avicel is generally less stable than endoglucanase under standard hydrolysis conditions [28], but an increase in stability does not necessarily lead to increased hydrolysis when PEG is added as the enzymes are still active and the total concentration of the final product (glucose) in the hydrolysis reaction increases over time even without PEG (Figure 1A,B).

### Effect of PEG on hydrolysis of Avicel via reduced water constraint

**Figure 3:**
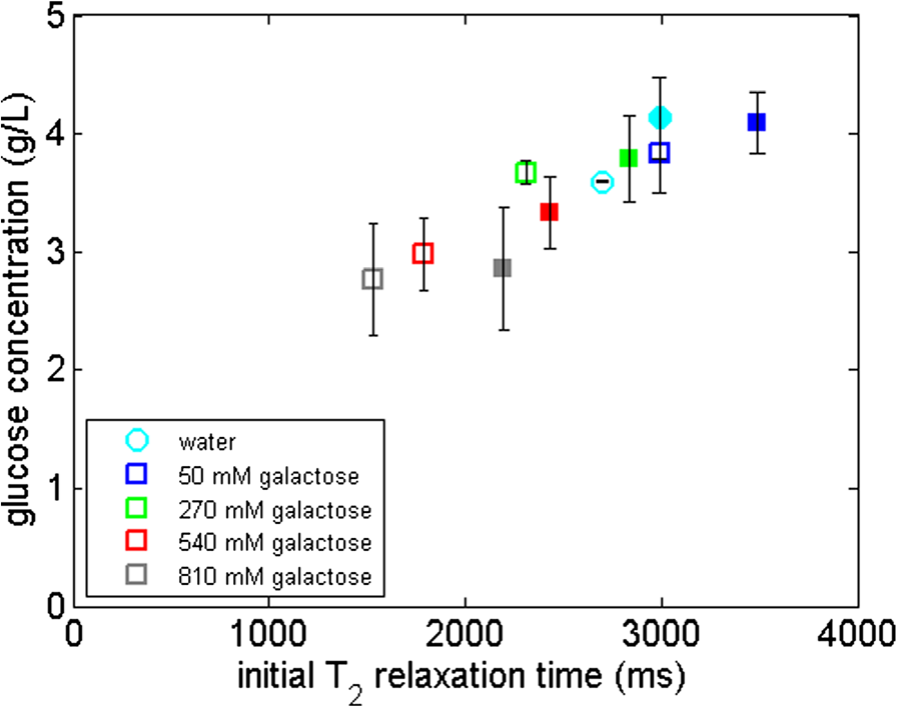
**Hydrolysis of 5% Avicel with monocomponent CBH I in galactose solutions at different concentrations without (open symbols) and with (solid symbols) PEG 3000.** The figure shows the relationship between the overall glucose production and the T_2_ relaxation time of the galactose solutions in which the hydrolysis was carried out. T_2_, spin-spin relaxation time.

To explore the possible link between yields and a PEG-induced reduction in water constraint/increased relaxation time, we added 10 to 150 g/L of galactose (a stereoisomer of glucose which does not inhibit cellulases and is not part of the hydrolysis reaction) during hydrolysis to introduce severe water constraint and tested the effect of PEG in these situations. These concentrations of galactose are based on lignocellulosic industrial production conditions, which can result in up to 150 g/L of glucose equivalent [8]. In Figure 3, we observe the following: as the concentration of galactose increases in the hydrolysis liquid phase, the T_2_ relaxation time of the solution goes down, consistent with an increase in water constraint; moreover, when PEG is added to highly constrained solutions of galactose, the T_2_ relaxation time goes up corresponding to a decrease in the water constraint; additionally, glucose production by the CBH I decreases when higher concentrations of galactose are present in the hydrolysis liquid phase, consistent with our earlier report that hydrolysis yields decrease when more solutes are present in hydrolysis [19]; lastly, the presence of PEG increases the hydrolysis yields for solutions with up to 270 mM galactose, but for even higher concentrations, there is no difference in yield with or without PEG, indicating a probable threshold for the boosting effect of PEG at this enzyme dosage. For hydrolysis using EG, the glucose production with and without PEG remained the same at all galactose concentrations (data not shown). These experiments had no cellobiose end-products; thus, β-glucosidase was not inhibited in the process even at >150-g/L galactose concentrations.

**Figure 4:**
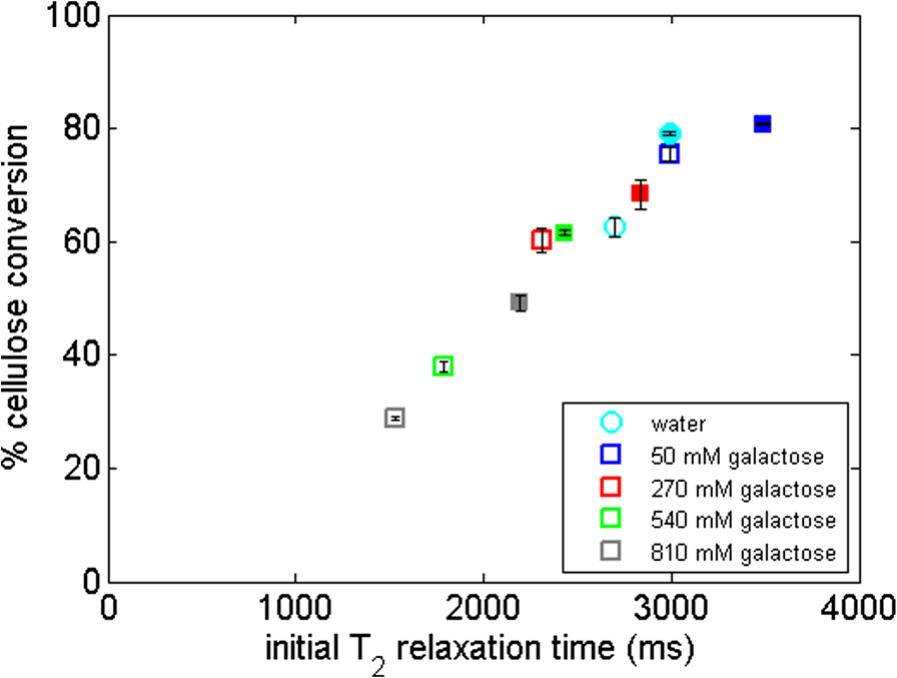
**Hydrolysis of 5% Avicel with Cellic CTec2 in galactose solutions at different concentrations without (open symbols) and with (solid symbols) PEG 3000.** The figure shows the relationship between the overall cellulose conversion and the T_2_ relaxation time of the galactose solutions in which the hydrolysis was carried out. T_2_, spin-spin relaxation time.

To better understand the role of CBH I-water interactions in the presence of PEG in more relevant industrial scenarios, we used a commercial cellulase mix, Cellic CTec2 (Figure 4). Here, we studied the correlation between the cellulose hydrolysis yield and the T_2_ relaxation time for a setup containing high concentrations of galactose (equivalent to a high-solids loading) which also constrains water in to an equivalent extent as glucose. As can be seen, there is a direct correlation between the T_2_ relaxation time of the hydrolysis liquid phase at the onset of hydrolysis and the cellulose conversion yield in the presence of galactose. For relaxation times higher than pure water, the overall correlation is less pronounced, maybe because water constraint is not limiting hydrolysis under these conditions.

**Figure 5:**
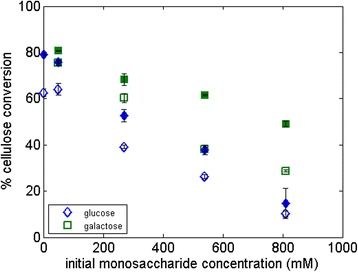
**Hydrolysis of 5% Avicel with Cellic CTec2, 1 wt% PEG 3000 and additional glucose or galactose.** The control samples (without PEG) are shown using open symbols.

Increasing the galactose concentration in hydrolysis decreased the overall conversion, which is also observed when additional glucose is added instead of galactose (Figure 5). Yields with additional galactose tend to be higher than glucose because glucose not only acts as a water-constraining molecule, but is also a powerful cellulase inhibitor. During the hydrolysis experiments shown in Figure 5, we did not observe any cellobiose at the end of the hydrolysis, showing that the β-glucosidases were still active in the presence of high concentrations of sugars. While PEG increases the cellulose hydrolysis rate and final glucose production, the cellobiose produced by CBH I is consumed regardless of whether or not PEG is present, consistent with the fact that the cellulases most inhibited by water constraint are those acting on the insoluble substrate and not the β-glucosidases [19]. This phenomenon is supported by the results of cellobiose hydrolysis using Cellic CTec2, where in the presence or absence of PEG, the cellobiose hydrolysis yield did not change (Figure 1C).

Looking back at the hydrolysis results obtained using monocomponent CBH I and EG in light of the correlation observed between PEG addition, increased yields and decreased water constraint, we suggest that the increased hydrolysis yield seen for CBH I in the presence of PEG has to do with the lower water constraint brought on by the surfactant. Thus, since kinetic and mechanistic studies reported in the literature suggest that the saccharification rate for CBH-type cellulases depends upon enzyme desorption rate [21-23,26], and since the presence of PEG increases the saccharification rate, then a link between enzyme desorption rate and the presence of PEG seems likely. Determining the influence of PEG on the kinetics of CBH I would be a subject of future work.

### Effect of different PEG molecular weights and concentrations on commercial cellulase mixtures

**Figure 6:**
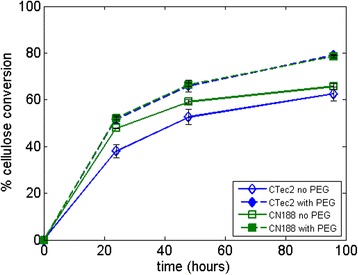
**Comparison of 5% Avicel hydrolysis using PEG 3000 between Cellic CTec2 and Celluclast 1.5 L with Novozym 188 supplementation (CN188) based on the same FPU activity (10 FPU per gram of Avicel).** The control samples (without PEG) are shown using open symbols. PEG, polyethylene glycol.

As PEG increases the activity of CBH I, it can also boost the glucose production of commercial cellulase preparations acting on Avicel. Figure 6 shows the hydrolysis of 5% Avicel in the presence of 1 wt% PEG using Cellic CTec2 or Celluclast 1.5 L with additional Novozym 188. The enzyme activity in the liquid phase was the same for both enzyme mixes, that is, the protein content was higher for Celluclast than for CTec2. With this setup, the addition of PEG gives approximately the same increase in conversion for CTec2 as for Celluclast with additional Novozym 188 supplementation, which suggests that PEG has a universal Avicel hydrolysis-boosting effect, as also shown by Ouyang *et al*. [17] and Zhang *et al*. [16]. Li *et al*. [14] have also shown a PEG-boosting effect on Avicel using Accellerase 1000 (Genencor, Palo Alto, CA, USA) with different enzyme loadings and PEG concentrations. Given that CBHs make up more than 70% of the *Trichoderma reesei* secretome [29] from which most cellulolytic enzyme mixtures are produced industrially [30], it is no surprise that the addition of PEG increases the hydrolysis yield for a range of enzyme preparations, which can be attributed to the PEG effect on CBH.

**Figure 7:**
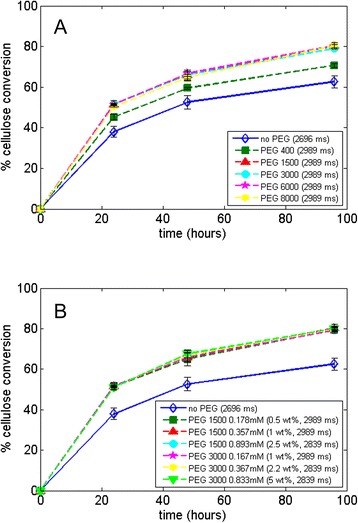
**Hydrolysis of Avicel using CTec2 at 5% dry matter at 24, 48, and 96 h with (A) different molecular weight of PEG (1%** ***w*** **/** ***w*** **) in water and (B) different concentrations (molar concentration and percentage weight per gram of cellulose) of PEG.** The control hydrolysis contained no PEG and was conducted in water (open symbols), and the T_2_ relaxation times of the hydrolysis liquid fraction at the onset of hydrolysis are shown in parenthesis next to the legend. PEG, polyethylene glycol.

We also tested different molecular weights of PEG (Figure 7A) and different PEG dosages (Figure 7B) on Avicel hydrolysis with CTec2. As seen in Figure 7A, if the same dosage is used, there is no significant difference in the glucose production by using solid PEG with different molecular weights in the 1,500 to 8,000 g/mol range, while liquid PEG with a molecular weight of 400 g/mol has a smaller impact. The hydrolysis results are in agreement with those reported by Ouyang *et al*. [17], who observed very small changes in the hydrolysis yield of Avicel with Celluclast 1.5 L supplemented with Novozym 188 in citrate buffer using PEG with different molecular weights (2,000 to 8,000 g/mol), while in a later study, Zhang *et al*. [16] observed a yield difference of 2% to 3% using different molecular weights of PEG (2,000 to 10,000 g/mol) under the same conditions, albeit using higher PEG loadings. However, other studies have found a more significant effect of PEG molecular weight on hydrolysis yields, most likely due to other factors including the different enzyme mix, PEG concentration, and the substrate being hydrolyzed. For example, when using Celluclast 1.5 L with Novozym 188 supplementation for the hydrolysis of either steam-pretreated spruce [4] or wheat straw [5], higher cellulose conversions were reported when PEG with higher chain lengths were used.

The reason why the molecular weight of PEG does not affect the boost in hydrolysis of Avicel can to a large extent be understood as an effect of water constraint. As shown in Figure 7A (legend), T_2_ relaxation time data of the hydrolysis liquid phase shows no difference between PEG molecular weights. Thus, an increase in PEG chain length does not affect water constraint. That yields are, however, different for liquid *vs*. solid PEG (400 g/mol *vs*. 1,500 to 8,000 g/mol) while their relaxation time is the same shows that LF-NMR spin-spin relaxation times depend on many factors and can only be used as an indicator of expected hydrolysis yields between sets of strictly comparable samples.

Regarding PEG dosage used, Figure 7B shows that PEG boosts Avicel hydrolysis with CTec2 compared to the control, but from 0.17 to 0.89 M (0.5 to 5 wt% of cellulose) of PEG in solution, the difference is minimal with respect to the increase in yield. LF-NMR data given in the figure also confirm the hypothesis that, as there is no difference in the hydrolysis liquid phase relaxation time between different concentrations of PEG, the glucose production remains the same. It is important to note, however, that the concentrations of PEG used in these studies are within a 1 to 5 wt% cellulose range, and results would probably differ if higher concentrations were used, as shown in Table 1. The relaxation time measurements show that PEG does not have an additive effect, that is, relaxation times do not increase with higher concentrations of PEG and eventually decrease when PEG concentrations are above 20% in solution. Such high-PEG concentrations, however, would be unrealistic for an industrial hydrolysis setting.

**Table 1:** **T**_**2**_ **relaxation times of PEG 3000 solutions**

**PEG 3000 concentration**		**Avicel 5 wt% equivalent (wt%)**	**T**_**2**_ **relaxation time (ms)**
**(wt% solution)**	**(mM)**		
0	0	0	2,696
0.0025	0.00833	0.05	2,818
0.005	0.0167	0.1	2,989
0.025	0.0833	0.5	2,989
0.05	0.167	1	2,989
0.125	0.417	2.5	2,839
0.25	0.833	5	2,839
0.5	1.667	10	2,839
1	3.333	20	2,696

## Conclusions

The addition of PEG to hydrolysis of Avicel has a positive effect on the hydrolysis yield. When present during hydrolysis using a CBH I, PEG can increase the overall hydrolysis yield up to 45%, whereas there is no significant increase in hydrolysis yields when using an EG with PEG. PEG does not seem to affect the performance of β-glucosidase, as yields remain the same with and without PEG during hydrolysis. Increased thermal stability and higher substrate accessibility in the presence of PEG have been ruled out as factors which could explain the higher activity of CBH I. We hypothesize that part of the increase in hydrolysis yield using a pure cellulosic substrate in the presence of PEG was due to the increased water availability upon addition of the surfactant. Based on LF-NMR data, the increase in hydrolysis yield was directly correlated to the increase in the relaxation time of the hydrolysis liquid phase.

This study stresses the fact that the properties of the hydrolysis liquid phase play a role in the overall hydrolysis yield aside from properties of the substrate and that the extent of water constraint in the hydrolysis liquid phase may either be increased or decreased compared to pure water depending on the solutes present. In a broader sense, this work illustrates that water constraint can be a mirror through which not only factors impeding cellulose saccharification can be studied, but also those boosting this process.

## Materials and methods

### Materials

PEG 400 (weight average molecular weight (M_w_) 380 to 420 g/mol), 1500 (M_w_ 1,400 g/mol), 3000 (M_w_ 3,000 g/mol), and 6000 (M_w_ 5,400 g/mol), were obtained from Merck, Darmstadt, Germany. Avicel, PEG 8000 (M_w_ 8,000 g/mol), D-glucose, D-galactose, and D-cellobiose standards were purchased from Sigma-Aldrich, St. Louis, MO, USA. Commercial cellulase mixtures Celluclast 1.5 L and Cellic CTec2, as well as the β-glucosidase mixture Novozym 188, were obtained from Novozymes A/S, Bagsværd, Denmark. Celluclast 1.5 L had a protein content of 127 mg/g, filter paper activity of 62 FPU/g, and a β-glucosidase activity of 15 U/g based on the following assays: filter paper activity was determined according to Ghose [31] and the β-glucosidase activity was measured using 5 mM *p*-nitrophenyl-β-D-glucopyranoside as substrate [32]. The protein content was measured using the Ninhydrin assay with BSA as a protein standard [33]. Cellic CTec2 had a protein content of 161 mg/g (120 FPU/g) and a β-glucosidase activity of 2,731 U/g. Novozym 188 had a protein content of 220 mg/g and a β-glucosidase activity of 231 U/g. The purified monocomponent enzymes CBH I from *Trichoderma longibrachiatum* (0.05 U/mg on *p*NP-lactoside), endo-β-glucanase from *Talaromyces emersonii* (64 U/mg on carboxymethylcellulose), and β-glucosidase from *Aspergillus niger* (52 U/mg on *p*NP-β-glucoside) were purchased from Megazyme, Wicklow, Ireland. All reagents and enzymes were used as received without further purification.

### Avicel hydrolysis with monocomponent cellulases and β-glucosidase

Fifty milligrams of Avicel were weighed in 2-mL screw-cap tubes and filled with diluted monocomponent enzyme solution in 50 mM citrate buffer at pH 4.8 for a total water-insoluble solids loading of 5% *w*/*w* (1-mL working volume). The first set of experiments consisted of CBH I (6 μL, or 1.2 mg protein/g Avicel) and β-glucosidase (6 μL, or 0.1 mg protein/g Avicel) (1:1 *v*/*v*), while in the second set, the CBH I was replaced by EG (6 μL, or 1.2 mg protein/g Avicel). The PEG 3000 concentration was 1 wt% (10 mg/g Avicel) and added to the diluted enzyme solution before adding to the substrate. Control samples without PEG were run in parallel. Hydrolysis was carried out in triplicate at 50°C in an Eppendorf Thermomixer (Hamburg, Germany) with shaking at 800 rpm. Time points were taken at 24, 48, 96, and 144 h. The hydrolyses were terminated by boiling at 100°C for 10 min, centrifuging at 13,200 × *g* and 5°C for 10 min, and filtering through a 0.45-μm syringe filter (Millipore, Billerica, MA, USA). The filtrates were stored at −20°C before HPAEC sugar quantification.

### PASC hydrolysis with monocomponent cellulases and β-glucosidase

The preparation of PASC was based on the procedure by Walseth [34] with a few modifications: 4 g of Avicel was suspended in 100 mL of phosphoric acid (85% *w*/*v*) at 1°C and magnetically stirred for 1 h. The mixture was then poured into 1,900 mL of ice cold water and kept at 1°C with further stirring for 1 h. The suspension was left stationary to allow the fibers to sink to the bottom of the flask while the supernatant was decanted. The suspension was washed four times with 2 L of MilliQ, two times 1% NaHCO_3_ solution to increase the pH to 5, and a further three times with water. The PASC then underwent a solvent exchange with 50 mM citrate buffer at pH 4.8 and stored at 1°C until further use. A full 24-h hydrolysis of PASC with excess CTec2 confirmed the cellulose content at 3.5% *w*/*v*.

PASC was hydrolyzed using the same monocomponent enzymes as described in the previous section using Avicel. PASC (0.5 mL) was pipetted into 2-mL screw-cap tubes with 10 μL of 1:1 cellulase:β-glucosidase mix (2 mg cellulase protein/g Avicel, 0.14 mg β-glucosidase protein/g Avicel) and a further 2.5 wt% PEG 3000. Control samples without PEG were also run in parallel. Hydrolysis was carried out in triplicates at 50°C in an Eppendorf Thermomixer with shaking at 800 rpm, where samples were analyzed for glucose yields after 5, 24, and 48 h. The samples were processed as described in the previous section with Avicel.

### HPAEC hydrolysis products quantification

Avicel and PASC hydrolysis products were quantified using an ICS5000 HPAEC system equipped with a PAD detector (Dionex, Sunnyvale, CA, USA). The separation was performed using a Dionex CarboPac PA1 anion exchange column, a 2 mm × 50 mm guard column and 2 mm × 250 mm analytical column. The column operated at a flow of 0.25 mL/min and maintained at 30°C. Peak separation for glucose and cellobiose was obtained by applying the following elution gradient and using fucose as an internal standard: water for 28 min, gradient to 0.1 M NaOH for 9 min and 0.1 M NaOH for 8 min, gradient to 0.2 M NaOH for 2 min and 0.2 M NaOH for 5 min, followed by column reconditioning with water for 8 min for a total run time of 60 min. Column elution was followed by a post-column addition with 0.2 M NaOH at 0.12 mL/min. For the analysis of oligosaccharides, the method was modified from Westereng *et al*. [35]. The elution gradient consisted of 0.1 M NaOH for the initial 10 min, a linear gradient to 0.1 M NaOH with 0.22 M NaOAc for 12 min, gradient to 0.1 M NaOH with 1 M NaOAc for 3 min, followed by a column reconditioning with 0.1 M NaOH for 15 min, for a total run time of 40 min.

### Cellobiose hydrolysis with Novozymes Cellic CTec2

D-cellobiose was dissolved in 100 mM sodium acetate buffer at pH 4.8 to make a concentrated solution of 100 g/L. The cellobiose was hydrolyzed using CTec2 with a protein loading of 0.064 mg/g of cellobiose. PEG 3000 was added to the reaction at 1, 2, and 5 wt% of cellobiose. The samples were mixed in an Eppendorf Thermomixer at 50°C and 800 rpm. Samples were prepared in triplicates, and time points were taken at 1, 2, 4, and 8 h. The hydrolysis was terminated by adding 72% H_2_SO_4_ and diluting to a final concentration of 3 mM H_2_SO_4_, which then the samples were centrifuged at 13,200 × *g* and 5°C for 10 min, filtered through a 0.45-μm syringe filter and analyzed using high-performance liquid chromatography (HPLC).

### Stability of monocomponent enzymes

Monocomponent enzymes (CBH I and EG) were diluted (6 μL, or 1.2 mg protein/g Avicel, in 1-mL 50 mM sodium citrate buffer at pH 4.8) with or without PEG 3000 (0.05% solution). The solutions were incubated at 50°C for 24 h in a rotating incubator, to which a monocomponent enzyme equivalent volume of β-glucosidase (6 μL, or 0.1 mg protein/g Avicel) was then added before adding to Avicel and hydrolyzing the substrate as described previously.

To test the monocomponent enzyme stability towards high-solute concentrations, four sets of galactose concentrations were added to the hydrolysis mix (5% Avicel) with and without PEG 3000 (1 wt% Avicel) using CBH I (1.2 mg protein/g Avicel) and EG (1.2 mg protein/g Avicel) with additional β-glucosidase supplementation (0.1 mg protein/g Avicel). PEG 3000 was first diluted in solution with the cellulases before adding to the substrate. The galactose concentrations were 50, 270, 540, and 810 mM. Hydrolysis was carried out as described previously, with control samples also run in parallel.

### Enzymatic hydrolysis of Avicel with Novozymes Cellic CTec2

Fifty milligrams of Avicel were weighed in 2-mL screw-cap tubes and filled with diluted enzyme solution for a total water-insoluble solids loading of 5% *w*/*w* (1-mL working volume). The enzymatic loading was 13 mg protein/g dry matter (approximately 10 FPU/g of Avicel). For hydrolysis with PEG, the loading was 1 wt% of the water-insoluble solids (10-mg/g Avicel), unless otherwise indicated. In a separate set of experiments, glucose and galactose were each used to spike the hydrolysates at 50, 270, 540, and 810 mM with and without PEG. Glucose was chosen as it is a known inhibitor of cellulases, while galactose, though not an inhibitor, is a stereoisomer of glucose. Hydrolysis was carried out as described above. Control samples (without enzyme) were also run in parallel.

### Enzymatic hydrolysis of Avicel with Celluclast 1.5 L/Novozym 188

A 5% *w*/*w* hydrolysate was prepared by measuring 50-mg Avicel in 2-mL screw-cap tubes and filled with diluted enzyme and PEG 3000 solution (1% weight per gram of dry matter) for a 1-mL working volume. A 5:1 weight mix of Celluclast and Novozym 188 at 10-FPU/g dry matter (19 mg protein/g) enzyme solution was used. The combined enzyme mixture had a filter paper activity of 76 FPU/g. Hydrolysis was carried out in triplicates at 50°C in an incubator mounted with an orbital shaker at 150 rpm. Time points were taken at 24, 48, and 96 h. The hydrolyses were terminated by centrifuging each sample at 13,200 × *g* and 5°C for 10 min, filtering through a 0.45-μm syringe filter, and analyzed using HPLC.

### HPLC sugar quantification

D-cellobiose, D-glucose, and D-galactose (standards obtained from Sigma-Aldrich, St. Louis, MO, USA) were quantified with a Dionex UltiMate 3000 (Dionex, Germering, Germany) equipped with a refractive index detector (Shodex, Minato, Tokyo, Japan). The separation was performed in a Phenomenex Resex ROA column at 80°C with 5 mM H_2_SO_4_ as eluent at a flow rate of 0.6 mL/min for 15 min. The results were analyzed using the Chromeleon software program from Dionex.

### LF-NMR T_2_ relaxation

LF-NMR measurements were performed on a Bruker mq20 minispec with a 0.47 T permanent magnet (equivalent to 20-MHz proton resonance frequency). The internal magnet temperature was 40°C. Samples were prepared by mixing 50, 270, 540, and 810 mM of galactose in separate vials with and without PEG. Then, 1 mL of sample was placed in 15 dia glass tubes and heated to 40°C, where the T_2_ relaxation times of the solutions were then measured using the CPMG pulse sequence. Each sample was run with 32 scans and a 5-s recycle delay containing 8,000 points and a pulse separation of 1.2 ms. The obtained relaxation curves were analyzed using the inverse Laplace transformation method CONTIN [36], where all the solutions gave a single T_2_ relaxation peak. The T_2_ reported are the peak maxima positions of this peak.

## Abbreviations

CBH: cellobiohydrolase
EG: endoglucanase
LF-NMR: low-field nuclear magnetic resonance
M_w_: weight average molecular weight
PASC: phosphoric acid swollen cellulose
PEG: polyethylene glycol
T_2_: spin-spin relaxation time
